# Oxidative stress in retinal pigment epithelium cells increases exosome secretion and promotes angiogenesis in endothelial cells

**DOI:** 10.1111/jcmm.12834

**Published:** 2016-03-21

**Authors:** Sandra Atienzar‐Aroca, Miguel Flores‐Bellver, Gemma Serrano‐Heras, Natalia Martinez‐Gil, Jorge M. Barcia, Silvia Aparicio, Daniel Perez‐Cremades, Jose M. Garcia‐Verdugo, Manuel Diaz‐Llopis, Francisco J. Romero, Javier Sancho‐Pelluz

**Affiliations:** ^1^ School of MedicineCatholic University of ValenciaValenciaSpain; ^2^ Wilmer Eye InstituteJohns Hopkins UniversityBaltimoreMDUSA; ^3^ Experimental Research UnitGeneral University Hospital of AlbaceteAlbaceteSpain; ^4^ Valencian Biomedicine Institute CSICValenciaSpain; ^5^ Department of PhysiologyUniversity of ValenciaValenciaSpain; ^6^ Department of Comparative NeurobiologyCavanilles Institute of Biodiversity and Evolutive BiologyUniversity of ValenciaPaternaValenciaSpain; ^7^ Department of SurgeryUniversity of ValenciaValenciaSpain

**Keywords:** exosomes, retinal pigment epithelium, oxidative stress, angiogenesis, VEGF receptors

## Abstract

The retinal pigment epithelium (RPE), a monolayer located between the photoreceptors and the choroid, is constantly damaged by oxidative stress, particularly because of reactive oxygen species (ROS). As the RPE, because of its physiological functions, is essential for the survival of the retina, any sustained damage may consequently lead to loss of vision. Exosomes are small membranous vesicles released into the extracellular medium by numerous cell types, including RPE cells. Their cargo includes genetic material and proteins, making these vesicles essential for cell‐to‐cell communication. Exosomes may fuse with neighbouring cells influencing their fate. It has been observed that RPE cells release higher amounts of exosomes when they are under oxidative stress. Exosomes derived from cultured RPE cells were isolated by ultracentrifugation and quantified by flow cytometry. VEGF receptors (VEGFR) were analysed by both flow cytometry and Western blot. RT‐PCR and qPCR were conducted to assess mRNA content of VEGFRs in exosomes. Neovascularization assays were performed after applying RPE exosomes into endothelial cell cultures. Our results showed that stressed RPE cells released a higher amount of exosomes than controls, with a higher expression of VEGFR in the membrane, and enclosed an extra cargo of VEGFR mRNA. Angiogenesis assays confirmed that endothelial cells increased their tube formation capacity when exposed to stressed RPE exosomes.

## Introduction

Exosomes are small vesicles, between 50 and 150 nm in diameter 1, released by a number of different cell types 2, 3, 4, 5. Invaginations in the late endosome limiting membrane produce a multivesicular body (MVB) full of intraluminal vesicles 6. Once the MVB is formed, it can fuse with the cell membrane releasing its cargo to the extracellular medium, which might subsequently interact with neighbouring cells 7. As such, exosomes can be found in many corporal fluids, including blood, saliva, breast milk and even aqueous humour 8, 9, 10, 11. Exosome cargo is comprised of genetic material and proteins, making these vesicles essential in cell communication 12.

The retinal pigment epithelium (RPE), a single cell layer that separates blood vessels from photoreceptors, accomplishes a pivotal role in retinal homoeostasis 13, 14, 15. As a result of its anatomical location and function, the RPE is continuously exposed to potential cell damage from oxidative stress, specifically because of reactive oxygen species (ROS) 16.

Neovascularization, or the formation of new blood vessels, is one of the most common hallmarks of blinding diseases, such as the proliferative forms of age‐related macular degeneration (AMD) and diabetic retinopathy (DR) 17, 18. Moreover, oxidative stress induces the formation of angiogenic factors, of which VEGF is most commonly known 19. Although VEGF has normal physiological functions in the retina 20, 21, elevated levels of secretion contributes to the development of new blood vessels, as seen in wet AMD and proliferative DR 22. VEGF‐A, the main isoform of the protein, acts through VEGFR‐1 and VEGFR‐2, VEGF receptors present in endothelial cells of blood vessels 23. Moreover, it was recently suggested that ROS induce VEGF secretion, thus resulting in enhanced neovascularization 24, 25. VEGF can be released by different retinal cells, such as RPE cells 26, 27, Müller cells 28 and choroid endothelial cells 29. VEGFR‐1 and ‐2 can be expressed in neural, glial and vascular cells 18. In fact, VEGFR‐2 levels were noted to be increased in vascular elements in patients suffering DR 30. Though it is well established that neovascularization is brought upon by high VEGF levels and increased VEGFR‐1 and VEGFR‐2 expression, the mechanisms of such overproduction in AMD or DR are still unidentified. Moreover, oxidative stress can be promoted through ethanol treatment 31, which has been demonstrated to enhance neovascularization in different tissues 32, including the choroid 33, 34.

It has been recently shown that certain exosomes are able to either promote or inhibit neovascularization 35, 36, but the mechanisms clarifying those events are poorly understood. The aim of the present study was to observe exosome secretion and alterations in exosomal cargo from stressed RPE cells and elucidate their potential role in angiogenesis. It has been suggested that damaged RPE exosomes secreted to the extracellular medium may carry a different cargo than healthy RPE exosomes. Levels of angiogenic factors, such as VEGF receptors, might be altered, which would thereby influence neighbouring endothelial cells. We have hereby demonstrated that the protein and mRNA exosomal cargo for VEGFR‐1 and VEGFR‐2 are increased when RPE cells are under stress, and that these exosomes may interact with endothelial cells influencing their angiogenic capability.

## Materials and methods

### Cell culture

Arising retinal pigment epithelium (ARPE‐19) human cell line was obtained from American Type Culture Collection (ATCC, Barcelona, Spain). ARPE‐19 cells were cultured as previously described 31, 37. Cells were used from 18 to 20 passages and cultured to 80–90% confluence in p100 culture well plates at a seeding density of 1 × 10^6^ cells/cm^2^. The use of sub‐confluent cell cultures might be a limitation for the direct applicability of the results to the physiological situation, though these sub‐confluent conditions are largely accepted in oxidative stress studies 25, 31, 37. Cells were treated for 24 hrs at different ethanol concentrations: 40 and 80 mM (absolute ethanol; Biosolve, Valkenswaard, The Netherlands). Subsequently, cells and supernatant were collected and preserved for future experiments.

Human umbilical vein endothelial cells (HUVEC) were isolated as described previously 38. Briefly, umbilical veins were perfused with 1% collagenase solution and incubated at 37°C for 15 min. Endothelial cells were recovered in specific endothelial growing medium (EGM)‐2 (Lonza, Cultek, Barcelona, Spain) and incubated at 37°C and 5% CO_2_.

### Exosome isolation and size distribution

Exosome isolation was performed modifying a protocol previously published 39. An extra ultracentrifugation at 40,000 × g for 30 min. was performed to remove microvesicles larger than 200 nm–1 μm, thus avoiding contamination in the final sample. The exosome pellet was stored at 4°C until further processing in PBS solution. For the microvesicle protein extraction, the pellet was sonicated – using a UP200S sonicator (Hielscher Ultrasonics, Teltow, Germany) – 6 cycles of 6 pulses (amplitude 75%), vortexed for 10 sec., and rotated by wheel at 4°C overnight. Subsequently, it was sonicated again 6 cycles of 6 pulses (amplitude 75%), vortexed for 10 sec., and stored at 4°C until further processing. Exosome identity was confirmed by the nanoparticle tracking system NanoSight NS300 following manufacturer protocols (Malvern Instruments, Malvern, UK).

### Western blot

An equal amount of protein from each sample (35 μg) was loaded. Protocols previously published were used 31. Membranes were incubated overnight at 4°C with antibodies against VEGFR‐1, VEGFR‐2 (1:500; Abcam, Cambridge, MA, USA), VEGF (1:500; Santa Cruz Biotechnology, Santa Cruz, CA, USA) and β‐actin (1:500; Santa Cruz Biotechnology) or α‐tubulin (1:500; Santa Cruz Biotechnology) as loading controls for cells. Finally, membranes were incubated for 2 hrs at RT in antimouse and anti‐rabbit IgG‐HRP (1:10,000; Santa Cruz Biotechnology). Bands were visualized with ECL (Pierce, Thermo Scientific, Rockford, IL, USA) and detected with Image Quant LAS‐4000 mini (GE Healthcare, Uppsala, Sweden). Protein levels were quantified by densitometry using ImageJ software (NIH, Bethesda, MD, USA). Protein expression intensity was normalized to the amount of loaded protein and plotted as a percentage of the control group.

### Electron microscopy

Cells were seeded in a Lab‐Tek chamber slide with eight wells (Nalge Nunc International, Naperville, IL, USA) and were fixed in 3% glutaraldehyde for 2 hrs at 37°C. Cells were post‐fixed in 2% OsO_4_ for 1 hr at room temperature and stained in 2% uranyl acetate in the dark for 2 hrs at 4°C. Finally, cells were rinsed in distilled water, dehydrated in ethanol and infiltrated overnight in Durcupan resin (Fluka, Sigma‐Aldrich, St. Louis, MO, USA). Following polymerization, embedded cultures were detached from the chamber slide and glued to araldite blocks. Ultrathin sections (0.06–0.08 μm) were prepared with the Ultracut and stained with lead citrate. Finally, grids were covered with handmade Formvar and photomicrographs were obtained under a transmission electron microscope FEI Tecnai G2 Spirit (FEI Europe, Eindhoven, Netherlands) using a digital camera Morada (Olympus Soft Image Solutions GmbH, Münster, Germany).

Exosome pellets were resuspended in PBS and ultracentrifuged at 120,000 × g for 70 min. at 4°C. After that, approximately 10 μg of the sample was resuspended in PBS on parafilm. The sample was fixed by depositing a drop of 2% PFA on the parafilm and placing the grid (Mesh with Formvar) on top of the drop. Negative staining was performed with 2% uranyl acetate. Photomicrographs were obtained using the transmission electron microscope previously described. Exosomes were identified under the microscope solely based on size and morphology.

### Flow cytometry

Exosomes were scrutinized by applying anti‐CD9 (Abcam), a well‐established exosome marker 39, 40, 41, using a FACScan flow cytometer (Beckman Coulter, Alcobendas, Spain). Similarly, antibodies anti‐VEGFR‐1 and ‐2 (Abcam) were used. Five hundred thousand events were collected for each sample. Results were analysed with BD FAC Suite software (Fullerton, CA, USA).

### Reverse transcription‐PCR and real‐time quantitative PCR

Following stress treatment, total RNA was obtained from ARPE‐19 cells and from derived exosomes using RNeasy Micro/Mini Kits (Qiagen, Hilden, Germany) according to manufacturer′s instructions. After extraction, total RNA was reversely transcribed (cDNA synthesis) using GoTaq PCR master mix (Promega, Fitchburg, WI, USA) under the following reaction conditions: 65°C for 5 min., 42°C for 60 min. and 70°C for 10 min. The cDNA was then subjected to a real‐time quantitative PCR (qPCR) using SYBR Green Supermix (Applied Biosystems, Carlsbad, CA, USA) in a Roche 234 LightCycler 480 PCR System. Primer sequences were: VEGFR‐1: Fwd 5′‐CCA CTT GAC ACT TTG ATC CCT G‐3; Rev 5′‐ATG CCC ATT GAC TGT TGC TTC‐3′; VEGFR‐2: Fwd 5′‐AGG GAC TTG GAC TGG CTT TG‐3′; Rev 5′‐AGG CTC CAG TGT CAT TTC CG‐3′). Each sample was analysed in triplicates and a cycle threshold (Ct) value of transcripts was determined in exponential phase using Light Cycler software 4.0. Finally, X‐fold change in mRNA levels was determined by applying 2^−ΔΔCT^ method. ΔCt values were calculated using endogenous control genes ribosomal S18 (Fwd 5′‐GTCTGTGATGCCCTTAGATG‐3′; Rev 5′‐AGCTTAT GACCCGCACTTAC‐3′) and GAPDH (Fwd 5′‐CAATGACCCCTTCATTGACC‐3′; Rev 5′‐GATCTCGCTCCTGGAAGATG‐3′) as referenced 42, 43.

### Vasculogenesis assay

Vasculogenesis was analysed in Matrigel (Becton Dickinson, Bedford, MA, USA) as previously described 44. After treating the cells in the absence or presence of RPE‐derived exosomes for 24 hrs, HUVEC (6 × 10^4^ cells/well) were recovered and seeded on Matrigel‐coated wells for 5 hrs. Matrigel was previously diluted with EGM‐2 media, FBS free and allowed to solidify for 30 min. at 37°C. Then, pictures were taken with a Nikon Eclipse‐Ti inverted microscope (Nikon, Tokyo, Japan).

## Results

### Isolation and characterization of ARPE‐19 cell‐derived exosomes

**Figure 1:**
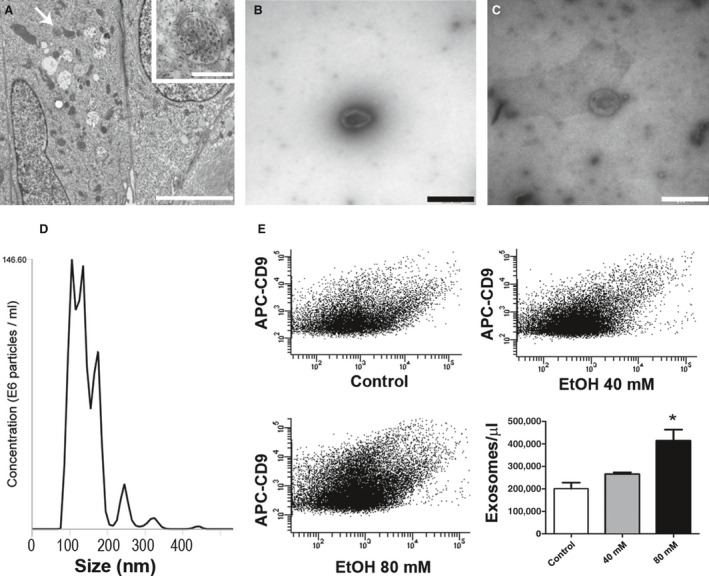
Exosome biogenesis. Formation of MVBs was observed in untreated cells (arrow in **A**) (higher magnification in the inset). Exosomes released from ARPE‐19 cells exhibit the classical morphology and size (50–150 nm). They were detected in extracellular medium from untreated cells (**B**) and from 80 mM ethanol‐treated cells (**C**). Flow cytometry exosome detection was performed targeting CD9. Size‐distribution analysis of exosomes was performed by Nanosight (**D**). Control ARPE‐19 cells released a lower number of exosomes (first plot in **E**) than those cells treated with 40 mM (second plot in **E**), and 80 mM (third plot in **E**). Quantification is shown in the bar graph. Scale bars represent 500 μm in A, 500 nm in the inset, and 100 nm in **B** and **C**. Values are expressed as mean ± S.E.M. (*N* = 3). Significance levels: *P* < 0.05 (*).

After isolation, exosome identity was confirmed by electron microscopy. Multivesicular bodies were observed within ARPE‐19 cells, before and after ethanol exposure (Fig. 1A). Exosomes were observed in the extracellular medium of both control cells and ethanol‐treated cells (Fig. 1B and C). Exosomes exhibited the typical characteristic morphology (cup‐shaped) and size (diameter between 50 and 150 nm). Exosome size was confirmed by the Nanosight tracking system (Fig. 1D).

For exosome counting, we used an anti‐CD9 antibody bound to a fluorescent antibody (APC). Ethanol‐treated ARPE‐19 cells released a higher amount of exosomes into the extracellular medium than non‐treated ones (Fig. 1E). In fact, such was observed to occur in a concentration‐dependent manner, as cells treated with 80 mM ethanol released a larger number of exosomes compared to those treated with 40 mM. The difference between control and treated group was only significant when ethanol concentration was 80 mM.

### VEGF and VEGFR‐1 from ARPE‐19 cells

**Figure 2:**
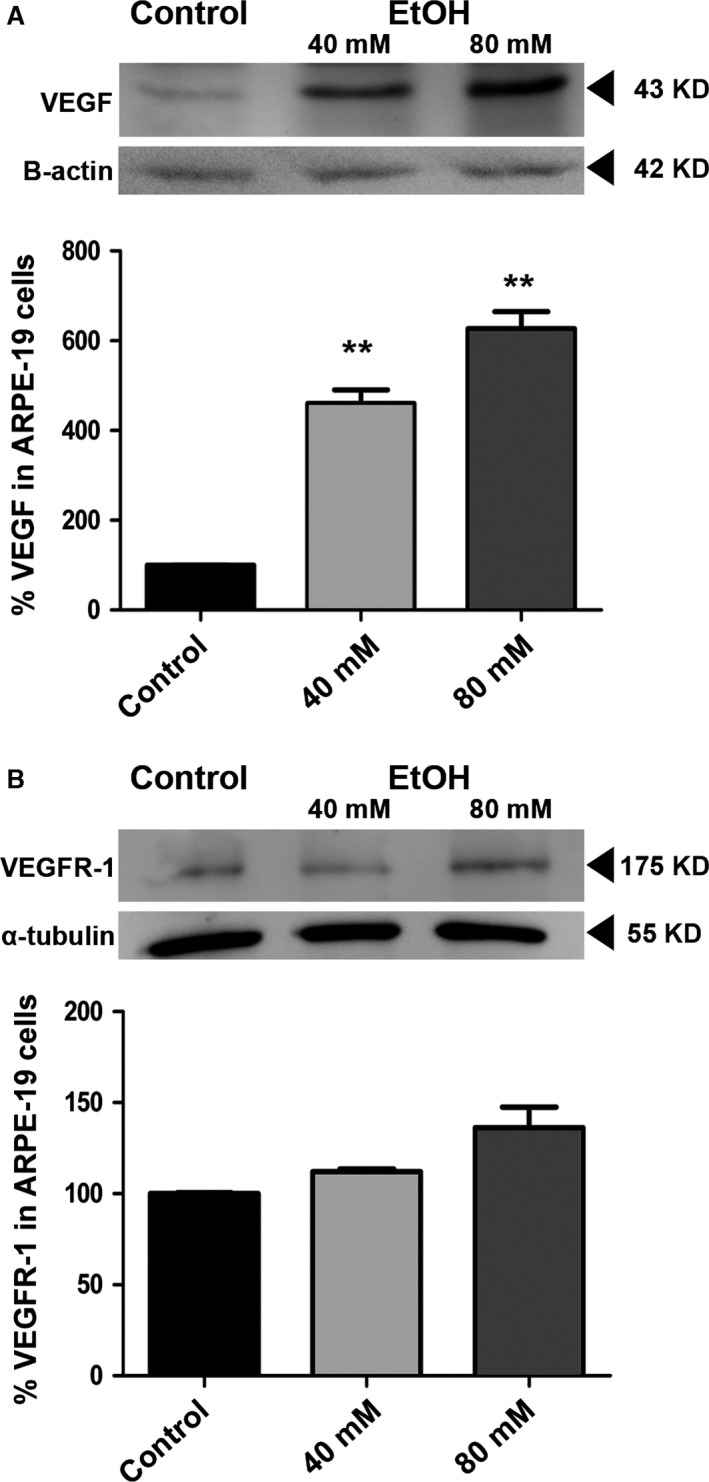
VEGF and VEGFR‐1 from ARPE‐19 cells. ARPE‐19 cells were incubated for 24 hrs in the absence (control) or presence of ethanol, and the result was analysed by Western blot. Non‐treated cells secreted a baseline amount of VEGF into the medium (first band in **A**), whereas ethanol‐treated cells secreted a significantly higher concentration of VEGF. Those observations were made using 40 mM ethanol (second band in **A**) and 80 mM ethanol (third band in **A**). Conversely, VEGFR‐1 did not show a significant change in release when cells were exposed to ethanol damage (**B**). All experiments were normalized to the loading control (B‐actin or α‐tubulin). Values are expressed as mean ± S.E.M. (*N* = 3). Significance levels: *P* < 0.01 (**).

A number of different cells are known to release VEGF into the extracellular medium, and RPE cells are among them. Control ARPE‐19 cells released baseline levels of VEGF (Fig. 2A, first band). After ethanol treatment, ARPE‐19 cells were noted to release a higher amount of VEGF glycoprotein. A significant fourfold increase in the release of VEGF was observed in cells treated with only 40 mM ethanol (Fig. 2A, second band). Apparently, VEGF release after oxidative insult by ethanol follows a concentration‐dependent manner, as it was observed that cells treated with 80 mM ethanol showed a significant sixfold increase in VEGF, when compared to untreated ARPE‐19 cells (Fig. 2A, third band).

It is well established that following a low stress insult, some cells can release an increased amount of VEGF receptors (VEGFR‐1 and ‐2). In this study, it was essential to verify whether the release of VEGF receptors following stress was a direct one or required the assistance of another mechanism, such as exosome secretion. Therefore, expression of VEGFR‐1 was studied in both ARPE‐19 cells and exosomes secreted by them. When VEGFR‐1 expression was tested in ARPE‐19 cells, no significant difference was noted between untreated and treated cells, either with 40 or 80 mM ethanol (Fig. 2B). Expression of VEGFR‐2 was below detectable levels.

### VEGFR‐1 and VEGFR‐2 in ARPE‐19 cell‐derived exosomes

**Figure 3:**
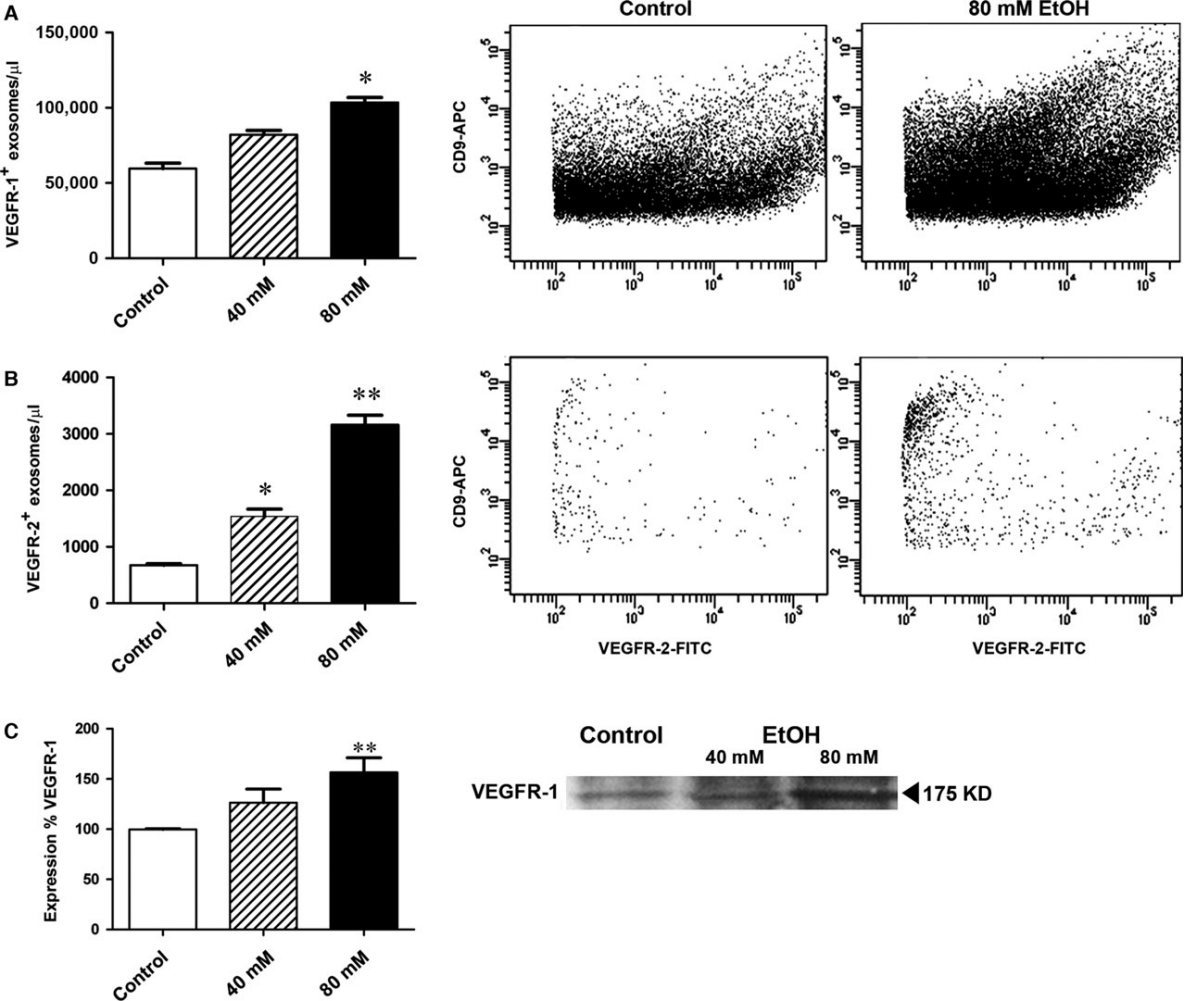
Effects of ethanol in exosomal VEGFRs content. ARPE‐19 cells were incubated for 24 hrs in the absence (control) or presence of different ethanol concentrations. Released exosomes were scrutinized by flow cytometry and Western blot. VEGFR‐1 protein was detected in control and ethanol groups, being significantly different at 80 mM of ethanol (**A**). VEGFR‐2 protein was also found in exosomes released from untreated cells, and its levels significantly rose in an ethanol concentration‐dependent manner (at 40 and 80 mM) (**B**). Presence of VEGFR‐1 in exosomes released by ARPE‐19 was also observed by Western blot, and results were in agreement with cytometry experiments (**C**). Values are expressed as mean ± S.E.M. (*N* = 3). Significance levels: *P* < 0.05 (*) and *P* < 0.01 (**).

Presence of VEGFR‐1 and ‐2 in exosomes was studied by means of flow cytometry and Western blot. A portion of the exosomes released from untreated ARPE‐19 cells presented VEGFR‐1 in their membranes (Fig. 3A, first bar). The number of VEGFR‐1‐positive exosomes showed a non‐significant increase when cells were treated with 40 mM ethanol (Fig. 3A, second bar). However, the number of VEGFR‐1‐positive exosomes increased significantly in the medium when cells were treated with 80 mM ethanol (Fig. 3A, third bar). A similar effect was noted when the expression of VEGFR‐2 was studied in exosomes. In untreated cells, the population that expressed VEGFR‐2 barely reached 700 exosomes per μL (Fig. 3B, first bar); when cells were exposed to 40 mM ethanol, VEGFR‐2‐positive exosomes doubled their number (Fig. 3B, second bar). Again, the population of VEGFR‐2‐positive exosomes seems to increase in a concentration‐dependent manner, as cells treated with 80 mM ethanol exhibited more than 3000 positive exosomes per μL (Fig. 3B, third bar).

To verify previous results, Western blot assays were performed. VEGFR‐1 expression was significantly different in the exosome population from ARPE‐19 cells formerly treated with 80 mM ethanol for 24 hrs (Fig. 3C), thus confirming the results observed in flow cytometry assays. VEGFR‐2 was not detectable by blotting analysis because of the limited detection level of the technique. VEGF presence within the exosomes was also examined, but it was under detectable levels in exosomes from both ethanol‐treated and untreated cells.

**Figure 4:**
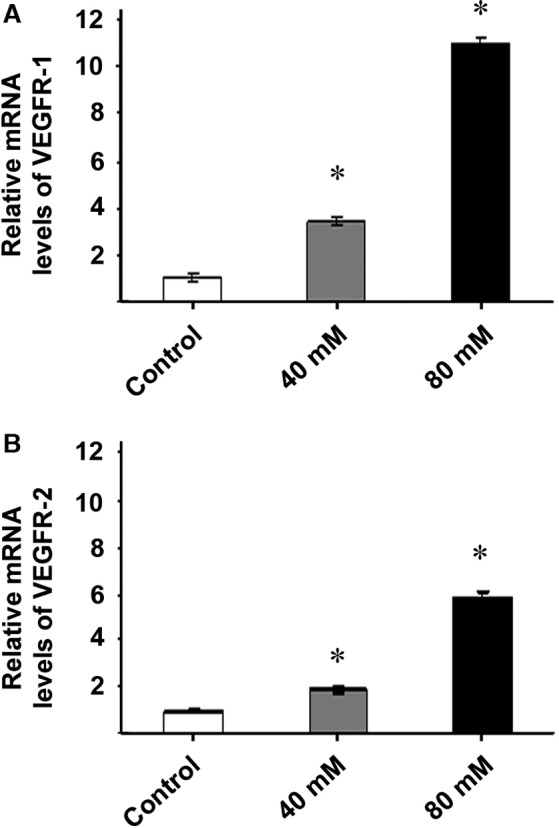
Exosomes released from ethanol‐exposed ARPE‐19 cells are highly enriched in VEGFR‐1 and VEGFR‐2 mRNAs. VEGFR‐1 and VEGFR‐2 transcript levels in exosomes released by ARPE‐19 were also analysed using reverse transcription assay followed by qPCR. ARPE‐19 cells were incubated for 24 hrs in the absence (control) or presence of different ethanol concentrations. RNA was extracted from the exosome released from ARPE‐19 cells. Presence of mRNA of VEGFR‐1 was found in control and ethanol groups, being its level significantly higher at 40 and 80 mM of ethanol (**A**). VEGFR‐2 mRNA was also detected in exosomes derived from untreated cells, and its amount increased in an ethanol concentration‐dependent manner (**B**). Values are expressed as mean ± S.E.M. (*N* = 3). *Significance level: *P* < 0.05.

We evaluated mRNA levels of VEGF receptors in exosomes derived from ARPE‐19 control cells and cells treated with various concentrations of ethanol. VEGFR‐1 and VEGFR‐2 mRNA levels were found to be markedly increased in the exosomes from treated cells, when compared with the control group. In the case of VEGFR‐1, mRNA levels in the exosomes were increased more than 10‐fold when cells where treated with 80 mM ethanol (Fig. 4A). Furthermore, VEGFR‐2 mRNA levels were increased when cells were treated with 40 mM and 80 mM of ethanol (Fig. 4B). Thus, exosomes from damaged retinal cells contain a higher level of VEGFR‐1 and VEGFR‐2 mRNA, and this phenomenon occurs in a dose‐dependent manner.

### Tube formation of endothelial cells treated with exosomes derived from ethanol‐treated ARPE‐19 cells

**Figure 5:**
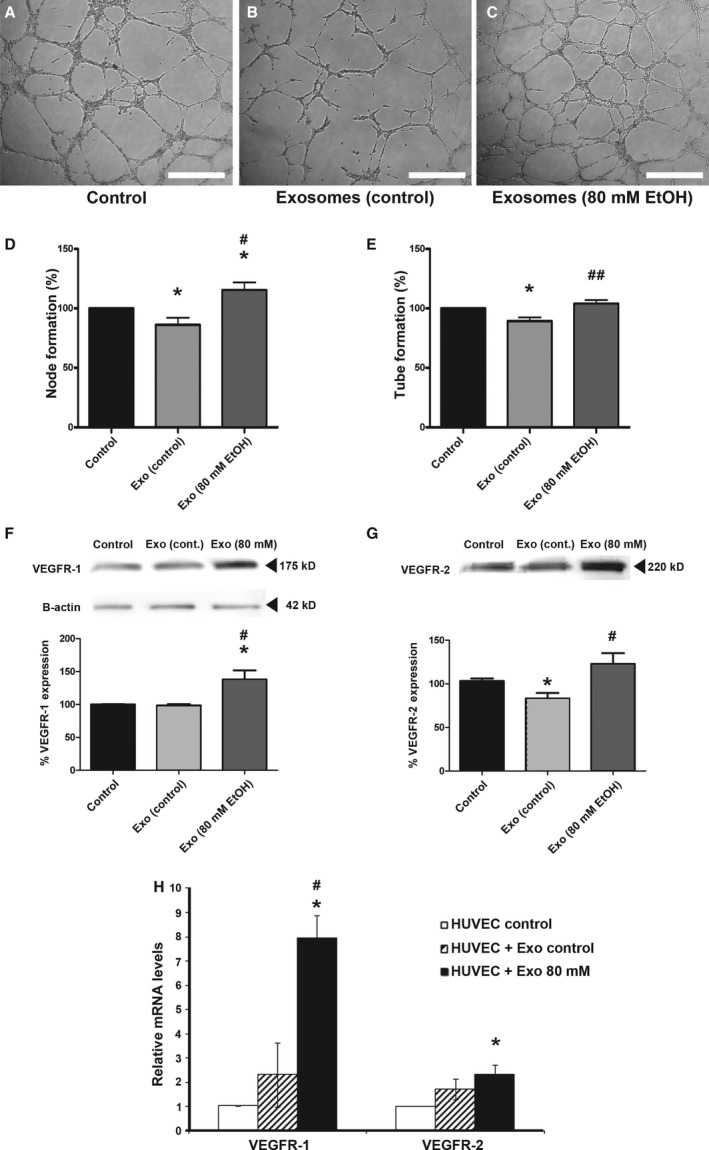
Vasculogenesis capacity of exosomes‐treated HUVEC cells. Exosome‐treated and ‐untreated HUVEC cells were seeded on Matrigel, showing specific tube formation after 5 hrs. Non‐treated HUVEC cells showed basal tube formation (**A**). Tube formation appeared less effective when HUVEC cells were treated with exosomes derived from non‐treated ARPE‐19 cells (**B**). When HUVEC cells were treated with exosomes derived from EtOH‐treated (80 mM) ARPE‐19 cells, tube building was more efficient (**C**). Node formation (**D**) and length of the new blood vessels (**E**) was quantified. After 5 hrs, HUVEC cells were pulled and VEGFR‐1 and VEGFR‐2 protein expression were studied by Western Blot (**F** and **G**). Loading control with B‐actin was performed for VEGFR‐1 and ‐2 (**F**). Amounts of mRNA were measured in HUVEC cells before and after exosome treatment, showing an enhancement when endothelial cells were treated with stressed exosomes (**H**). Scale bars: 500 μm. Values are expressed as mean ± S.E.M. (*N* = 3). Significance levels, when compared to control: *P* < 0.05 (*); when compared to Exo (control): *P* < 0.05 (#) and *P* < 0.01 (##).

The formation of tubes by endothelial HUVEC cells occurred without external influence, however components in the culture medium are able to accelerate or inhibit the process. When HUVEC cells were seeded in Matrigel and treated with the appropriate medium for 5 hrs, they migrated, assembling in a precise configuration to create tubes which at the end of the process will materialize as brand new blood vessels (Fig. 5A). After 5 hrs in culture, HUVEC cells treated with healthy RPE exosomes seemed to slow the tube formation process (Fig. 5B). Surprisingly, damaged RPE exosomes had a completely different influence, when used to treat HUVEC cells. Actually, these exosomes appeared to influence HUVEC cells, accelerating tube formation (Fig. 5C). Those results were quantified by observing the formation of the nodes and the length of the new tubes formed (Fig. 5D and E respectively). The amount of VEGFR‐1 and VEGFR‐2 in endothelial cells was significantly enhanced when exosomes from stressed ARPE‐19 cells were applied to the medium (Fig. 5F and G). Relative amounts of VEGFR mRNA in HUVEC cells were also examined (Fig. 5H). Compared to untreated cells, those treated with stressed exosomes showed an eightfold increase of VEGFR‐1 mRNA and a twofold increase of VEGFR‐2 mRNA.

## Discussion

Previous data indicate that ARPE‐19 cells, a human RPE cell line, when exposed to low‐medium stress conditions, do not degenerate but rather undergo increased autophagy 31, enhanced VEGF release 45, and heightened exosome secretion 46. Moreover, low ethanol concentrations promote oxidative stress in ARPE‐19 cells 31, 37. Moderate levels of ethanol are also known to induce VEGF expression in cardiomyocytes 47, tumour cells 48 and RPE cells 45.

Recent studies have shown that a number of cell types, including RPE cells can influence neighbouring cells by the release of exosomes 49, 50. In fact, exosomes from retinal astrocytes seem to reduce vessel leakage in a model of AMD, whereas RPE‐originating exosomes do not stop the new vessels from leaking 35. Moreover, it has been proposed in another AMD model that exosomes released by ageing RPE cells are able to increase autophagy in neighbouring cells, and that this can contribute to drusen formation 51. In parallel, another group demonstrated that exosomes derived from mesenchymal stem cells inhibited neovascularization by down‐regulating VEGF expression 52.

ARPE‐19 cells were treated with low concentrations of ethanol (40 and 80 mM). As seen in the results section, ARPE‐19 cells consistently released exosomes positive for VEGFR‐1 and ‐2. However, when those cells were treated with ethanol, they secreted a larger quantity of exosomes, many of which contained membrane‐bound VEGFR‐1 and ‐2. Interestingly, when RT‐PCR and qPCR experiments were performed, higher amounts of VEGFR‐1 and ‐2 mRNA were observed within the vesicles.

Therefore, when RPE cells are exposed to low amounts of ethanol, they release a high quantity of exosomes containing VEGF receptors in their membrane, and VEGFR‐1 and ‐2 mRNA within the exosome. When RPE‐derived exosomes interact with neighbouring endothelial cells, there would be an increased potential for membrane fusion and, thereby, incorporation of VEGF receptors in the new cell membrane 53. Exosomes may also release genetic cargo into the cytosol of the second cell ensuring the delivery of mRNA to its genetic machinery 54, making possible an augmentation in the expression of VEGF receptors.

Human umbilical vein endothelial cells in culture tend to build tubes by themselves 44. Surprisingly, a significant delay in blood vessel formation was noticed when healthy RPE exosomes were added to the medium. Stressing this fact, levels of VEGFR‐2 were reduced in the membrane of HUVEC cells after the treatment (Fig. 5G). On the contrary, when HUVEC cells were influenced by damaged RPE exosomes, tube formation occurred more effectively. Furthermore, HUVEC cells influenced by stressed exosomes showed higher levels of VEGF receptors, both protein and mRNA (Fig. 5F–H). These facts strongly indicate that healthy RPE exosomes (under physiological conditions) inhibit choroidal tube formation, whereas those released from stressed ARPE‐19 cells promote vasculogenesis/angiogenesis. In fact, it has been observed that exosomes secreted from hypoxic cells promote angiogenesis 55. This angiogenic effect might be because of the extra cargo of proteins and mRNA contained in the exosomes, as RPE‐derived exosomes do not contain detectable levels of VEGF in any case. In conclusion, exosomes maintain certain control over tube formation, and it somehow depends on the homoeostatic state of the releasing cell.

## Conflicts of interest

The authors have no conflict of interest to declare.

## Author contribution

JMB, FJR and JSP designed the research. SAA, MFB, GSH, NMG, SA, DPC and JMGV performed the research. SAA, NMG, MDL and GSH analysed the data. JMB, FJR and JSP wrote the paper.
